# Sensors and Devices Based on Electrochemical Skin Conductance and Bioimpedance Measurements for the Screening of Diabetic Foot Syndrome: Review and Meta-Analysis

**DOI:** 10.3390/bios15020073

**Published:** 2025-01-26

**Authors:** Federica Verdini, Alessandro Mengarelli, Gaetano Chemello, Benedetta Salvatori, Micaela Morettini, Christian Göbl, Andrea Tura

**Affiliations:** 1 Department of Information Engineering, Università Politecnica delle Marche, Via Brecce Bianche, 12, 60131 Ancona, Italy; f.verdini@univpm.it (F.V.); a.mengarelli@univpm.it (A.M.); m.morettini@univpm.it (M.M.); 2 CNR Institute of Neuroscience, Corso Stati Uniti 4, 35127 Padova, Italy; gaetano.chemello@cnr.it; 3 Unit of Medical Genetics, IRCCS Istituto Giannina Gaslini, Via Gerolamo Gaslini, 5, 16147 Genoa, Italy; benedettasalvatori@gaslini.org; 4 Department of Obstetrics and Gynaecology, Medical University of Vienna, Währinger Gürtel 18-20, 1090 Vienna, Austria; christian.goebl@meduniwien.ac.at

**Keywords:** diabetic foot, skin conductance, bioimpedance, early screening, home care, neuropathy, type 2 diabetes

## Abstract

Diabetic foot syndrome is a multifactorial disease involving different etiological factors. This syndrome is also insidious, due to frequent lack of early symptoms, and its prevalence has increased in recent years. This justifies the remarkable attention being paid to the syndrome, although the problem of effective early screening for this syndrome, possibly at a patient’s home, is still unsolved. However, some options appear available in this context. First, it was demonstrated that the temperature measurement of the foot skin is an interesting approach, but it also has some limitations, and hence a more effective approach should combine data from temperature and from other sensors. For this purpose, foot skin conductance or bioimpedance measurement may be a good option. Therefore, the aim of this study was to review those studies where skin conductance/bioimpedance measurement was used for the detection of diabetic foot syndrome. In addition, we performed a meta-analysis of some of those studies, where a widely used device was exploited (SUDOSCAN^®^) for foot skin conductance measurement, and we found that skin conductance levels can clearly distinguish between groups of patients with and without diabetic neuropathy, the latter being one of the most relevant factors in diabetic foot syndrome.

## 1. Introduction

Diabetic foot syndrome is a multifactorial disease. Indeed, diabetic foot pathophysiology includes different etiological factors, with peripheral neuropathy being particularly relevant [1]. Nonetheless, diabetic foot syndrome often lacks early symptoms; in addition, its prevalence is increasing [2]. These aspects make diabetic foot syndrome particularly ominous, and this explains the attention received by this syndrome in recent years, also mirrored by the updates to the relevant guidelines [3,4]. In fact, it was reported that appropriate screening programs could even lead to a 75% reduction in amputations [5]. On the other hand, such a percentage indicates that the problem of diabetic foot-related amputations is far from being solved.

In this context, sensors and devices for the screening, at an early stage, of diabetic foot syndrome (or the risk of such a syndrome) are particularly relevant. The relevance would be even higher if such devices were intended for personal, domiciliary use, rather than for ambulatory or hospital use. This would increase the probability of patients at risk for diabetic foot syndrome having such a device at home and hence performing frequent screening. Unfortunately, such a situation is far from being realized. To our knowledge, currently, the only type of device that can be commonly used for diabetic foot screening at home is a simple digital thermometer. Indeed, on one hand, many diabetic patients have a blood glucose meter at home, but such a device can only indicate a generic increase in the risk for diabetic foot syndrome as well as for other diabetes-related complications when glycemic levels worsen. Therefore, having a different device at home, devoted to specifically detecting a possible increased risk for diabetic foot syndrome, remains an open challenge. As regards digital thermometer-based measures, several studies have shown that a temperature differences between the two plantar regions may be an early biomarker of diabetic foot syndrome, as also outlined by several review studies focusing on the opportunities offered by thermal analysis of the feet in the context of diabetic foot syndrome [6,7,8,9,10,11,12,13]. The thermal approach has the advantage of simplicity of the measurement and, when performed by a simple digital thermometer, the advantage of low cost. However, such an approach also has several drawbacks, since the thermometer detects the temperature at a single spot on the skin, and, in addition, it is difficult for the patient to always repeat the measurement at the same spot, which affects reproducibility over time. In addition, there are issues related to the reliability of the approach, since the foot temperature may vary depending on several factors, not necessarily related to the presence of diabetic foot syndrome. Besides, for some patients, when elderly and/or obese, it is difficult to reach the plantar zone of the foot, which causes difficulties in the use of a thermometer in that area. It should also be acknowledged that there are some (though very few) devices performing thermal measurements in the feet that should overcome some of the limitations of the simple digital thermometer, such as the SmartMat™ [14,15,16], the Thermoscale [17,18], and the TempStat^®^ [19] devices. However, according to the manufacturer’s web site [16] the SmartMat™ device appears to be available only under a one-year prescription, and it is unclear whether the patient can then decide to purchase it and at what cost; besides, it is unclear whether the device is also available outside the US market. As regards the Thermoscale, the device seems available for personal direct purchase from a distributor’s web site [18], but information on the device is scarce (including information about the manufacturer), and at any rate, the device appears available only in the UK market. Finally, it is unclear whether TempStat^®^ is currently on the market. In addition to the above-mentioned devices, which have a scales-like format, there are a few having a socks-like or shoes-like formats, such as Siren Socks [20,21,22], SmartSox [23], and Bonbouton’s smart shoes [24,25], the latter aiming to integrate plantar pressure measurements with the thermal measurements. However, as regards Siren Socks, it is unclear under what conditions and at what cost the device is available, whereas SmartSox and Bonbouton’s smart shoes appear to only be available at a prototype level. Thus, even with regard to foot thermal assessment only, a satisfactory solution is not yet available.

In summary, in our opinion, a device for screening for diabetic foot with proper characteristics for personal domiciliary use is not yet available. Of note, we believe that such a device should not only be at low (not too high) cost and based on a simple measurement approach, but it should also ensure fair accuracy and precision and easy use by any type of patient. For this purpose, the correct approach may be combining different types of sensors in a single device. One type of sensor should certainly be for temperature detection, but some other types of sensors should be considered, including sensors for electrochemical skin conductance or, more generally, for bioimpedance measurements. Indeed, such sensors are typically simple and low cost (similarly to the temperature sensors), and their information may in fact complement that of the temperature sensors for improved accuracy in the early detection of diabetic foot syndrome or in the prediction of a remarkable risk for it. Of note, nowadays, it is relatively straightforward to combine information from different sensors and sources because of the opportunities offered by the modern machine learning/artificial intelligence technologies that have already proven to be useful in the context of diabetic foot syndrome, as documented by some review studies [26,27,28,29,30,31,32,33].

Thus, the aim of this review is to analyze studies where skin conductance/bioimpedance measurements were performed with application to the screening of diabetic foot syndrome (more precisely, for the identification of one or more of the above indicated etiological factors at the origin of the syndrome). To our knowledge, this is the first review study on skin conductance/bioimpedance measurement in the context of diabetic foot syndrome. In addition, we will also perform a meta-analysis over a subset of the studies included in the review, which is another novelty of our study.

## 2. Materials and Methods

### 2.1. Scientific Literature Search Strategy

The scientific literature was searched in PubMed by one author. Afterward, a second author checked the search strategy and agreed with the selection made by the first author.

Following testing of different PubMed search strings, we identified this final string:

diabet*[tw] AND (foot*[ti] OR feet*[ti] OR neuropath*[ti] OR polyneuropath*[ti] OR poly-neuropath*[ti] OR pheripher*[ti] OR nerv*[ti] OR autonom*[ti] OR skin*[ti] OR epiderm*[ti] OR derm*[ti] OR cutan*[ti]) AND (conductiv*[ti] OR conductan*[ti] OR resistiv*[ti] OR (resistance*[ti] AND (eletric*[ti] OR skin*[ti] OR epiderm*[ti] OR derm*[ti] OR cutan*[ti])) OR electroderm*[ti] OR electro-derm*[ti] OR electric*[ti] OR bioelectric*[ti] OR bio-electric*[ti] OR impedance*[ti] OR bioimpedance*[ti] OR bio-impedance*[ti] OR dielectric*[ti] OR electromagnet*[ti] OR electro-magnet*[ti]) NOT stimulat*[ti]

According to PubMed guidelines, “ti” searches in the article title, whereas “tw” (“text word”) allows searching in all main fields of PubMed records, i.e., in the title, abstract, MeSH terms, and some additional fields. The symbol “*” allows searching for all variations of a word root, e.g., diabetes, diabetic, etc.

**Figure 1:**
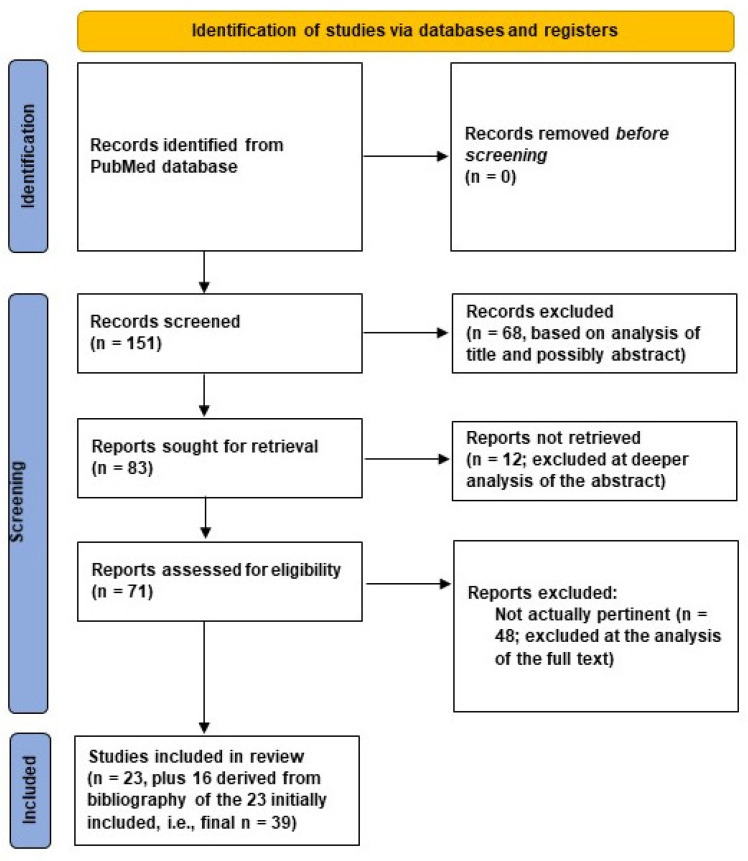
PRISMA flow diagram of the literature search strategy.

The literature search provided 151 items (last check: 14 May 2024). After analysis of these items, we finally selected 23 articles as appropriate for our study. The related PRISMA flow chart is depicted in Figure 1. Not pertinent articles were excluded after one of the following steps: (i) analysis of the article title (and, possibly, a quick look at the abstract); (ii) analysis of the whole abstract; or (iii) retrieval of the article full text and related analysis. Articles were deemed not pertinent if they did not actually include diabetic patients (despite mentioning diabetes and, especially, the diabetic foot issue somewhere in the article) or, when including diabetic patients (and specifically addressing the diabetic foot issue), they did not report conductance or bioimpedance measures of interest for the foot condition.

It also has to be noted that from the reference list of the 23 selected articles, we identified another 16 articles relevant for our study, for a total of 39 articles included. In this review, we did not consider articles from Congress proceedings or articles not in the English language.

In the following sections, we summarize the main information of the selected studies, with a focus on the main physiological/clinical goals and outcomes and on the methodological aspects related to skin conductance/bioimpedance measurement. The review is organized into two sections: the first is related to studies where a specific instrument was used, being very common for this type of study, i.e., the SUDOSCAN^®^, Impeto Medical SAS, Issy Les Moulineaux, France; the second section describes studies where other sensors and devices were used. In each section, articles are reported in chronological order. Synthetic information about each study is reported in Table 1 and Table 2.

### 2.2. Statistics for Meta-Analysis

For those studies with SUDOSCAN^®^, where the device measurements were evaluated against a reference method for the detection of one or more factors related to diabetic foot syndrome (typically, neuropathy), we performed a meta-analysis.

Specifically, those studies based on the use of SUDOSCAN^®^ for detecting sudomotor dysfunction were analyzed according to the PICOS framework [34]. The primary aim was to assess the validity of the measurement of transcutaneous electrochemical skin conductance (ESC) from the feet for identifying peripheral autonomic neuropathy in diabetic patients.

The inclusion criteria for selecting the studies for the meta-analysis were as follows: (i) studies had to include participants both with and without diabetic neuropathy; (ii) neuropathic conditions had to be documented and verified using standard methods for characterizing this pathology; (iii) selected studies needed to report ESC measurements from the feet using the SUDOSCAN^®^ system.

The meta-analysis was conducted using Metalab software (1.0 Version) developed by Mikolajewicz et al. [35]. Mean values and standard deviations of ESC measures were extracted from each study group. The mean difference between neuropathic and non-neuropathic groups (the latter serving as the control) was computed, and the size of the combined effect was analyzed using 95% confidence intervals (CIs). Statistical heterogeneity among studies was assessed using the inconsistency test and measured with H^2^ and I^2^ parameters [35]. A two-sided *p*-value less than 0.05 was considered indicative of a significant difference. Heterogeneity was categorized as low, moderate, or high based on I^2^ values: less than 25% for low, between 25% and 50% for moderate, and greater than 50% for high [35].

Based on the inconsistency test results, a fixed-effects or a random-effects model was chosen for analyzing the combined effect. The fixed-effects model assumed that all studies were measuring the same underlying effect, while the random-effects model considered that each study was estimating a different true effect, with the combined effect representing the average of these true effects. In the latter case, the variance comprises both intra-study variance (due to experimental sampling error) and inter-study variance (due to variability in true effects).

## 3. Studies with the Use of SUDOSCAN^®^

**Figure 2:**
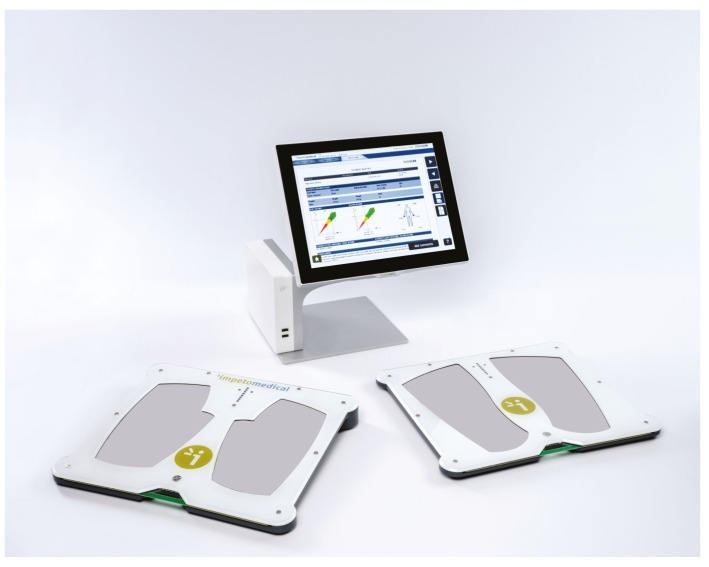
The SUDOSCAN^®^ system: the device consists of a computer and four electrodes on which patients place their hands and bare feet.

SUDOSCAN^®^ is a commercial device and is indicated on its web site (https://www.sudoscan.com/; last checked: 7 January 2025) as being a medical device that has been cleared in many countries, including Europe, the USA, and China (see Figure 2). From a technical point of view, we summarize here some technical information that is in fact illustrated in the indicated web site. The device consists of a computer receiving signals from a plate with four electrodes, on which the patients place the palm of their hands or the soles of their feet (where the density of sweat glands is the highest). The device evaluates sudomotor function of hand palms or feet soles by providing an electrical stimulus to the sweat glands and hence measuring the sweat glands’ ability to release chloride ions in response to the stimulus. It is reported that the underlying technology is based on the principles of the electrochemical reaction between chloride (present in sweat) and nickel (a component of the stainless-steel electrodes of the device). A low direct voltage (≤4 volts) is applied, generating a current relative to the chloride ion flow supplied by the sweat glands and ducts. At the indicated low voltage, the stratum corneum of the skin acts as a capacitor, leaving the sweat ducts as the only channels for the transmission of the chloride ions. Thus, an ESC measure is derived for each hand or each foot, based on the current generated at the voltage supplied.

In the following paragraphs of this article section, we report some information on those studies that used SUDOSCAN^®^ in the context of diabetic foot syndrome. Summary information about these studies is reported in Table 1.

In 2010, Mayaudon et al. [36] conducted a study to evaluate the effectiveness of the EZSCAN device (later called SUDOSCAN^®^) in assessing impairment in sudomotor function, a clinical manifestation of diabetic peripheral neuropathy (DPN). In fact, sudomotor dysfunction in diabetic people is indicative of damage to the small nerve fibers that control sweat glands, which is a common issue in neuropathy. The aim was to determine if EZSCAN, which measures electrochemical skin conductance (ESC) using reverse iontophoresis and chronoamperometry, could provide a sensitive, specific, and reproducible method for detecting neuropathy. The study involved 133 type 2 diabetic patients (mean age 58.9 ± 12.1 years, diabetes duration 14 ± 10 years) and 41 healthy controls (mean age 25.5 ± 6.4 years) with no risk factors for diabetes. Participants placed their hands and feet on nickel electrodes, where six combinations of 15 different low DC voltages were applied for 2 min. ESC was calculated from the resulting voltage and generated current. The diagnostic performance of ESC was analyzed using the receiver operating characteristic (ROC) curve, and reproducibility was assessed through Bland–Altman analysis. Results indicated that ESC was significantly lower in diabetic patients (hands: 53 ± 16 µS, feet: 67 ± 14 µS) compared to controls (hands: 68 ± 16 µS, feet: 80 ± 7 µS; *p* < 0.0001). The method showed a sensitivity of 75% and a specificity of 100%. Reproducibility coefficients of variation were 15% for hand measurements and 7% for feet. The study concluded that

In 2012, Yajnik et al. [37] conducted another study to evaluate SUDOSCAN^®^ (i.e., EZSCAN) in assessing sudomotor function for the screening of neuropathy in type 2 diabetic patients. The aim was to compare SUDOSCAN^®^ with traditional measures of peripheral and cardiac neuropathy. The study involved 265 type 2 diabetic patients (149 males and 116 females, 53.08 ± 9.07 years, with a diabetes duration of 9.32 ± 7.09 years). Participants were tested for neuropathy symptoms, vibration perception threshold (VPT), and cardiac autonomic neuropathy (CAN). Sudomotor function was evaluated with SUDOSCAN^®^, where lower ESC values indicated sudomotor dysfunction. SUDOSCAN^®^ involved the placement of hands and feet on nickel electrodes where low direct current was applied for 2 min, and ESC was calculated based on the electrochemical reaction between sweat chloride and the electrodes. Results showed that lower ESC at the feet was significantly associated with increased neuropathy symptoms and higher scores on physical abnormalities. Specifically, lower ESC was associated with increased VPT (*p* < 0.01), indicating decreased vibration perception, and with a higher number of abnormal CAN results (*p* < 0.05). ESC was related to postural blood pressure fall (*p* < 0.05), but not heart rate variability tests, suggesting that sudomotor dysfunction correlates more with sympathetic rather than parasympathetic abnormalities. The study confirmed that SUDOSCAN^®^ is a feasible, non-invasive, and rapid method for assessing sudomotor dysfunction in diabetic patients that can be easily integrated into routine clinical practice to alert physicians to the presence of peripheral nerve and cardiac sympathetic dysfunction.

An evaluation of the efficacy of SUDOSCAN^®^ in detecting DPN in patients with diabetes mellitus (DM) was also conducted by Casellini et al. in 2013 [38]. The study involved 83 DM patients (20 type 1, 63 type 2) with or without peripheral neuropathies compared with a database of 210 healthy controls (HCs). The study showed that hands and feet ESC is lower in patients with DPN. Specifically, feet ESC was lower in patients with painful DPN compared to patients with nonpainful DPN (52.8–3.6 vs. 68–6.6, *p* < 0.05). On the other hand, type 1 and type 2 DM patients with DPN had similar feet and hands ESC. SUDOSCAN^®^ proved to be a sensitive tool to detect neuropathy in patients with DM, as mirrored by the ROC curve analysis (for feet, area under the curve of 0.86, with sensitivity of 78% and specificity of 92%). Test–retest reliability was excellent for the feet, with a correlation coefficient of 0.814 (*p* < 0.0001).

In 2014, Smith et al. [39] investigated the potential usage of ESC assessment for the diagnosis of distal symmetric polyneuropathy (DSP). In this study, a total of 97 individuals were enrolled and sorted into a group of 55 DSP patients and a group of 42 control subjects, and the ESC was measured through the SUDOSCAN^®^ device. Among the DSP patients, the majority had diabetes and/or idiopathic neuropathy. All the volunteers placed their hands and feet on stainless steel plates, and a direct current potential, less than 4 V, was applied during a 120 s epoch. The ESC was measured as the ratio between the resultant current and the constant direct current stimulus. DSP patients showed significantly lower values of ESC for both feet and hands with respect to controls. The ESC provided the same diagnostic conclusion of the intraepidermal nerve fiber density (IENFD) in 58% of the cases, whereas the agreement between ESC and nerve conduction examination was 67%. Similar diagnostic capability of ESC and IENFD was also confirmed by similar area under the curve (AUC) values (0.761 and 0.752, respectively), indicating that the SUDOSCAN^®^ device is promising for DSP diagnosis.

In 2015, a population of 296 patients with diabetes mellitus was enrolled for a cross-sectional study performed by Sheshah et al. [40]. They noticed that in daily practice, the monitoring of ESC is limited because the standardized methods generally used are complex and require specialized equipment. In their study, the ESC was assessed through SUDOSCAN^®^, which guarantees rapid, non-invasive, and robust assessment of sudomotor function. Indeed, sudomotor dysfunction (SMD) can occur when autonomic neuropathy is associated with diabetes mellitus, and the dryness of foot skin resulting from SMD, can increase the risk of foot ulcers in these patients. Thus, the early recognition of SMD signs can constitute a relevant issue for preventing complications. Patients enrolled were clinically evaluated to recognize painful neuropathic symptoms and peripheral neuropathy and to score the risk of ulcers. Measurement of ESC was performed on both the hands and feet, and sudomotor dysfunction was diagnosed based on exceeding the threshold values already defined in literature. Among the participants, 46.3% did not show SMD signs, while 28.4% had moderate and 25.3% had severe SMD. A significant positive correlation was recognized between fasting blood glucose or HbA1c and patients with severe SMD. Moreover, the authors observed a decrement in the ESC value measured at the feet as the severity of peripheral neuropathy (and hence the risk of ulceration) increased. Thus, the study showed that ESC measurement can be introduced to screen for SMD and peripheral neuropathy and hence identify patients with ulcer risk.

Again in 2015, Selvarajah et al. [41] tested the effectiveness of SUDOSCAN^®^ as a screening device for DPN. In this study, a total of 70 subjects were enrolled; 45 of them were affected by type 1 diabetes, and 25 were healthy controls. The presence of DPN was assessed according to the criteria of the American Academy of Neurology, using nerve conduction studies and clinical examinations. Patients were also split into groups with CAN, with subclinical CAN, and without CAN. The ESC was significantly lower in patients with neuropathy with respect to both non-neuropathic and healthy controls, whereas no differences were detected between non-neuropathic and healthy subjects. For DPN, the sensitivity of the ESC taken from the feet was 87.5% with a cut-off of ≤77.0 µS, while the specificity was 76.2%, with an AUC of the ROC of 0.85. Patients with CAN showed significantly lower ESC values with respect to no-CAN patients and healthy individuals for both feet and hands. Performances of foot ESC for the identification of CAN and no-CAN patients was in general lower with respect to DPN recognition, with an AUC of 0.66 and a sensitivity and specificity of 60.0% and 76.0%, respectively. Authors concluded that SUDOSCAN^®^ can be considered a valid screening tool for DPN.

In 2016, the work by Leclair-Visonneau et al. [42] dealt with the assessment of sweat function as a method for investigating sympathetic nerve fibers that innervate sweat glands. The authors tested the feasibility of ESC assessment as a measure of sweat function for children. For this purpose, the SUDOSCAN^®^ system was employed. A total of 100 children (male/female ratio: 45/55) aged between 2 and 17 years were enrolled for the study. Volunteers had to remain still for 3 min with bare hands and feet on instrumented plates of the SUDOSCAN^®^ system. The outcomes showed almost constant ESC values for the examined age range, and no significant correlations were reported between age and the ESC measured from both hands and feet. Further, no significant differences were found between male and female individuals. Quite similar average values of ESC for hands and feet were reported as normative values in children, i.e., 80.1 ± 6.6 µS and 81.9 ± 6.2 µS, respectively, together with a limited asymmetry between right and left limbs (3.0 ± 3.5% for hands and 2.4 ± 3.9% for feet). Normative values reported for children were similar to those reported for adults, indicating that sudomotor nerve maturation happens during childhood, since ESC depends upon sweat gland density. Beyond providing normative ESC values in children, this work also supports the value of ESC for evaluating small fiber neuropathy, since ESC has been previously tested for diabetic peripheral polyneuropathy to detect dysautonomia.

Again in 2016, Vinik et al. [43] provided normative values for ESC for healthy subjects, taking into account several characteristics such as age, height, weight, sex, glycemic profile, lipid profile, and ethnicity. The aim was to find possible relationships between the above-mentioned characteristics and the level of ESC for both hands and feet in order to unveil the existence of confounding factors that can affect the reliability in the identification of sudomotor dysfunction, often observed in diabetic patients. In the 2016 study, a total of 1350 healthy individuals were considered, and cardiovascular diseases, symptoms, or medical conditions related to peripheral neuropathy were grounds for exclusion. The effect of Hispanic ethnicity was studied on Mexican subjects, whereas effects of race on ESC was evaluated on 117 African American, 96 Indian, and 120 Chinese participants, by comparison with the white population. The ESC was measured by the SUDOSCAN^®^ device, and the subjects had to maintain their hands and feet in contact with stainless steel electrodes for a 2 min testing period. For 570 white individuals (age lower than 70 years), the median ESC was 76 µS, with a 5th–95th percentile interval of 62–86 µS for hands and 83 µS with 75–89 µS for feet, with high significant correlation (>0.95, *p* < 0.0001) between left and right limbs. Weak negative correlations (not lower than –0.2) were found between ESC and age, and no correlations with the body mass index (BMI) were found. Significantly lower ESC values were found for African American, Indian, and Chinese individuals with respect to the white population for both hands and feet (*p* < 0.0001). No sex differences were observed. The ESC also showed very limited variations between a measure taken at rest and a second measure taken after exercise, supporting the use of ESC as a screening solution when a period of rest before measurements is not guaranteed.

In 2017, the effectiveness of using ESC for early detection of DPN was investigated by Goel et al. [44] through comparison with the vibration perception threshold (VPT) test and the diabetic neuropathy symptoms (DNS) score. For this study, 523 patients with type 2 diabetes were enrolled. A total of 110 patients were included in the DPN group, and 413 patients were included within a non-DPN group. The ESC was measured through the SUDOSCAN^®^ system, whereas the VPT was assessed through a biothesiometer at different body sites such as the first, third, and fifth metatarsals, the great toe, the medial arch, the heel, and the dorsum of the feet. For the ESC, sudomotor dysfunction was recognized with values lower than 60 µS. DNS values equal to or higher than 1 indicated the presence of abnormalities. The DPN group showed higher values of VPT and lower ESC with respect to the non-DPN group, and abnormal ESC values had the highest prevalence in the DPN group (85%) with respect to both VPT (72%), and DNS (54%). For diagnosis purposes, ESC and VPT provided comparable specificity (85% and 90%), but ESC showed a higher sensitivity (85% versus 72%), whereas DNS presented the lowest values (60% specificity and 52% sensitivity). In addition, the AUC for ESC was the highest among the three diagnosis methods, i.e., 0.88 compared to 0.84 for VPT and 0.60 for DNS. Thus, the outcomes of the study suggested feet ESC as a viable method for assessing DPN if compared to two other well-acknowledged methods, such as VPT and DNS, by using 60 µS as a threshold.

Later on, Krieger et al. [45] in 2018 compared the performance of the SUDOSCAN^®^ system for DPN diagnosis with the sudomotor axon reflex test (QSART). In their study, they considered a total of 47 patients with type 2 diabetes. Among them, 27 were affected by DPN. In addition, 16 healthy controls were enrolled. The QSART was measured by the Q-Sweat system, which is based on the measure of the sweat response as the increase in humidity. Capsules were attached to the forearm and shank, with the outer chamber filled with 10% acetylcholine, and the inner chamber detects moisture from sweat changes. The measurement protocol was based on 300 s of baseline recording, 300 s acetylcholine iontophoresis at 2 mA, and 300 s of post stimulation. The ESC results were significantly lower for DPN patients with respect to control subjects, whereas no significant differences were detected between DPN and no-DPN groups or between the no-DPN group and healthy individuals. On the other hand, patients and controls showed no significant differences for sweat volume and response latency measured by the Q-Sweat system for both upper and lower limbs. ESC showed good capabilities in DPN identification, with AUC values for the ROC of 0.705 for feet and 0.714 for hands. Sweat volume and latency results were not able to be distinguished between the DPN and no-DPN groups. Thus, the study confirmed that ESC measured with SUDOSCAN^®^ has the potential to be considered a valid screening tool for DPN, outperforming QSART for this kind of task.

Again in 2018, a study by Mao et al. [46] aimed at investigating the effects of bilirubin on functions of small nerves by evaluating the association between serum bilirubin and peripheral nerve dysfunction. For this aim, the ESC was measured in 265 patients (102 females) affected by type 2 diabetes. Blood samples were collected for assessing serum total bilirubin, conjugated bilirubin level, and unconjugated bilirubin, measured after at least 8 h of fasting, using the AU640 analyzer (Olympus corp. Japan). The SUDOSCAN^®^ device was used for ESC assessment, and a threshold of 60 µS of mean foot ESC was used for diabetic peripheral neuropathy diagnosis. Overall, patients were sorted into three groups, i.e., with mild, medium, and severe neuropathy signs. Based on the ESC, 148 out of 265 patients were affected by peripheral neuropathy, with statistically significant different values of ESC for both foot and hand (44.98 ± 19.89 versus 75.68 ± 9.05 for the foot and 47.75 ± 17.54 versus 73.27 ± 9.67 for the hand, in peripheral neuropathy and non-peripheral neuropathy groups, respectively). Significant correlations (Spearman coefficient) were found between the ESC values at hands and feet and the total bilirubin and between ESC values and unconjugated bilirubin, but not with conjugated bilirubin. Multiple linear regression analysis revealed that higher total bilirubin was independently associated with higher ESC values, as happened for the unconjugated bilirubin but not for the conjugated bilirubin. Based on these results, the authors advocated for a possible protective role of high bilirubin levels in the development of diabetic neuropathy.

Furthermore, in 2018, the study by Shivaprasad et al. [47] aimed to provide normative values for ESC among healthy Indian participants and assess the potential influence of parameters such as age, sex, and body mass index (BMI) on ESC measurements. The analysis was conducted by measuring ESC through SUDOSCAN^®^ in the hands and feet of 217 healthy participants aged 18–75 years and with BMI < 25 kg/m^2^. Mean ESC values for the hands and feet were equal to 68.9 ± 13.1 and 71 ± 12.9 µS, respectively. It was observed that ESC values from the right and left hands and feet significantly correlated (r = 0.9, *p* < 0.0001), as well as ESC values of the hands and feet (r = 0.94, *p* < 0.0001). As for the effects of age, a weak but significant inverse correlation with ESC was observed for the hands (r = 0.02, *p* = 0.01) and for the feet (r = 0.12, *p* < 0.0001), whereas no significant difference was observed between male and female participants. Eventually, no significant correlation was observed with BMI for hands or feet. The authors concluded that the normative values for Indians found in this study are lower than those previously reported for Caucasians.

Another study by Shivaprasad et al. in 2018 [48] investigated, in 523 diabetic patients (18–65 years of age), the factors associated with abnormal ESC and early DPN. The sensory-function evaluation included assessments of pinprick, vibratory, light touch, and joint position senses, while the motor-function evaluation included assessments of each patient’s bilateral reflexes and muscle power. Neuropathy disability score (NDS) was adopted to determine the presence of DPN. Patients with NDS ≥ 6 were considered DPN positive, while those with NDS < 6 were considered DPN negative. The ESC (again assessed with SUDOSCAN^®^) was monitored at the hands and feet, and based on the ESC value, the patients were subdivided into two groups, one composed of subjects with foot ESC values lower than 60 μS, which was considered sudomotor dysfunction positive, and the second with higher foot ESC values, who were considered sudomotor dysfunction negative. It was found that sudomotor dysfunction was prevalent in patients with peripheral neuropathy, and it was significantly related to higher values of age, foot abnormalities, diabetes duration, and glycated hemoglobin.

Still in 2018, Duchesne et al. [49] performed a study to investigate the relationship between ESC and small nerve fiber density in skin biopsy in patients with polyneuropathy. The study was conceived as a retrospective single-center study, involving a total of 63 patients in whom foot ESC was evaluated through the SUDOSCAN^®^ device. Each patient was classified as having abnormal (below the fifth percentile) or normal ESC values. Moreover, proximal and distal leg skin samples were obtained through skin biopsy to assess autonomic sweat gland and somatic intraepidermal nerve fiber density (SGNFD and IENFD, respectively). A significant though weak correlation was found between ESC and SGNFD (r = 0.49, *p* = 0.0005) and between ESC and IENFD (r = 0.42, *p* = 0.0005). It was also found that several patients with abnormal ESC values had a normal SGNFD, thus suggesting that some unmyelinated fibers are present but not functional. On the basis of the obtained results, the authors concluded that ESC may be useful for the assessment of the function of fibers innervating sweat glands. However, mechanisms other than the loss of such fibers may be responsible for sweat gland dysfunction in polyneuropathies.

In one further study in 2018, Binns-Hall et al. [50] evaluated the feasibility of a combined eye, foot, and renal screening for the early diagnosis of diabetic distal polyneuropathy (DPN). To evaluate polyneuropathy, the authors recruited 236 people who attended the retinal screening for respectively performing two clinical and two instrumental assessments. The clinical assessment consisted of an evaluation with the Toronto Clinical Neuropathy Score and with the 10 g monofilament test. The instrumental assessment was performed through a hand-held device that measures sural nerve conduction velocity and amplitude (DPN-check) and through SUDOSCAN^®^ for sudomotor function. The diagnostic utility of the last two devices was assessed against the Toronto Clinical Neuropathy Score, considered as the gold standard. The prevalence of polyneuropathy was, respectively, 30.9% and 14.4% when evaluated by the first two clinical methods. Using DPN-check, the prevalence was 51.5%, while with SUDOSCAN^®^, it was 38.2%, and the results of both the devices significantly correlated with the gold standard.

In 2019, a by Cabré et al. [51] was aimed at the early detection of diabetic peripheral neuropathy (DPN), identified as the most common complication of diabetes, often leading to diabetic foot syndrome. The authors also stated that early unmyelinated C-fiber dysfunction is one of the typical signs of DPN and the first clinical manifestation of dysfunction indicating sudomotor eccrine gland impairment. The study focused on the measurement of ESC (indicated as dermal electrochemical conductance, DEC) as a quantitative expression of the sudomotor reflex for the screening of DPN. The study included 197 people, ranging from normal glucose tolerance to type 2 diabetes (T2D). Specifically, the study aimed to determine the performance of DEC for the screening of DPN as compared with the Semmes–Weinstein 5:07 monofilament (MFT), electromyography (EMG), the Neuropathy Disability Score (NDS), and the Douleur Neuropathique 4 Questions (DN4). DEC was measured by the SUDOSCAN^®^ device. It was found that DEC had high specificity (up to 95%) when compared to MFT, EMG, NDS, and DN4, each assumed to be a possible reference for DPN identification, but the sensitivity was low. The authors concluded that DEC is feasible in typical clinical practice and may be a good screening test for neuropathy in populations with prediabetes and T2D. However, such a conclusion may be questionable based on the findings of that study, since in our opinion, high sensitivity (although with possible low specificity) may be more appropriate for screening purposes.

Carbajal-Ramirez et al., again in 2019 [52], examined the effectiveness of SUDOSCAN^®^ in detecting neuropathy by comparing the measurements of ESC at the hands and at the feet with scores from the Michigan Neuropathy Screening Instrument (MNSI). A total of 221 patients were enrolled and divided into two groups of patients: those less than 5 years and those at least 5 years or more from diagnosis of type 2 diabetes. For MNSI, participants underwent a physical examination that included evaluation of skin changes, infection, muscle stretch reflexes, vibration sensation using a 128 Hz “diapason” on both feet, and pressure perception using a 10 g monofilament. The instrumental examination with SUDOSCAN^®^ was performed at the hands and at the feet. In both groups, patients with neuropathy according to MNSI had lower conductance in the hands and feet. Using MNSI as a reference, hands or feet abnormal ESC (<60 μS and 70 μS respectively) had a sensitivity of 97% and a positive predictive value of 87% to detect neuropathy in patients with longer diabetes duration. In patients with diabetes duration of less than 5 years, the sensitivity of abnormal hands or feet ESC for detection of neuropathy was 91%, while the positive predictive value was 88%.

In 2020, a study by Gatev et al. [53] investigated the ESC measure and the role of asymmetry parameters (i.e., right and left sides) from SUDOSCAN^®^ in the diabetic foot in relation to diabetes duration in an attempt to determine when sudomotor dysfunction initiates. The study considered a population of 165 individuals with different diabetes durations spanning from newly diagnosed diabetes (group 1, n = 31), longer diabetes duration and neuropathy (group 2, n = 33), presence of diabetic foot ulcer (group 3, n = 20), and, eventually, prediabetes (the latter acting as the control group, n = 81). Mean ESC for the hands and feet was measured, as well as mean asymmetry (calculated as the percentage of the difference between right and left ESC values divided by the higher of the two ESC values). The authors observed significantly different values for the above-mentioned parameters between group 3 and the control group. Moreover, a tendency to decrease (statistical significance was not always reached) was observed in mean hands and feet ESC values spanning from group 1 to group 3; in parallel, a tendency to increase was observed in asymmetry percentage from group 1 to group 3. The authors also showed that feet asymmetry was able to discriminate individuals with diabetic foot with very high sensitivity and specificity, and, to this purpose, a cut-off value of >9.5% was established, thus concluding that feet asymmetry could act as a marker of early damage.

Again in 2020, D’Amato et al. [54] proposed a study to investigate the diagnostic performance for diabetic cardiovascular autonomic neuropathy (CAN) and DPN of the ESC measure, especially when coupled with an appropriate questionnaire. CAN was diagnosed with the cardiovascular reflex tests (CARTs, assumed as the gold standard for the diagnosis of CAN) and then further assessed with a questionnaire named COMPASS 31. DPN was assessed using other questionnaires (the Michigan Neuropathy Screening Instrument Questionnaire, MNSI-Q, and the Michigan Diabetic Neuropathy Score, MDNS), in addition to the analysis of the vibration perception threshold (VPT) measured by a biothesiometer at the hallux dorsum and at the lateral malleolus and detection of the warm (WTT) and cold thermal perception thresholds (CTT), assessed with the TSA-II Neurosensory Analyzer (Medoc, Ramat Yishai, Israel) at the dorsum of both feet. An appropriate combination of the results from MNSI-Q, MDNS, VPT, WTT, and CTT allowed the diagnosis of DPN. Sudomotor function was then assessed through ESC measured at the hands and feet using SUDOSCAN^®^. ESC measures were considered abnormal if <50 μS and <70 μS for hands and feet, respectively. One hundred and two participants with diabetes, thus at risk for diabetes foot syndrome, were studied. The most relevant findings were that ESC and the COMPASS 31 questionnaire had sensitivities of 83% and 75% for CAN (though with somehow lower specificity) and specificities of 67% and 65% for DPN (though with somehow lower sensitivity), respectively. When combining ESC and COMPASS 31, the sensitivity for CAN increased to 100%, and the specificity for DPN increased to 89%.

In 2022, a study by Lin et al. [55] investigated the application of SUDOSCAN^®^ for screening microvascular complications (among which is DPN) in patients with T2D in China. To this aim, ESC of the feet and hands was evaluated in a population of 515 patients (274 males and 241 females, age between 23 to 89 years) with T2D and, on the basis of ESC values, each patient was assigned to the normal or the abnormal group (considering 60 μS as the cut-off value). Patients were also screened for DPN through the vibration perception thresholds (VPT) test. The results showed that hands and feet ESC was significantly and negatively correlated with VPT. Patients with ESC in the abnormal group (<60 μS) were found to have a 5.63-fold increased likelihood of having DPN than those with ESC in the normal group. Moreover, a binary logistic regression analysis was performed, and it was found that ESC (together with VPT) is an independent factor for DPN. Analysis of the ROC curve resulted in values for the area under the curve equal to 0.71 for both hands and feet ESC; cut-off values were 73 μS (sensitivity 61%, specificity 71%) and 61 μS (sensitivity 79%, specificity 65%) for hand and feet ESC, respectively. The authors concluded that SUDOSCAN^®^ could be used as an effective tool in primary health care for early screening of microvascular complications, among which is DPN.

Again in 2022, Zhao et al. [56] explored the utility of ESC, in combination with the ankle-brachial index (ABI), for diagnosis and prediction in patients with T2D of the peripheral artery disease (PAD) condition, which is a known etiological factor for diabetic foot syndrome. A total of 183 Chinese T2D patients were enrolled and stratified into three groups, one including uncomplicated T2D patients (group 1), one for patients with diabetic peripheral neuropathy (group 2), and one for patients showing PAD (group 3). Sudomotor function was assessed in all patients using a SUDOSCAN^®^ device. Specifically, the measured parameters were mean hands ESC (HESC, μS), mean feet ESC (FESC, μS), hands asymmetry (HASYM, %), feet asymmetry (FASYM, %), and cardiovascular autonomic neuropathy risk. Multivariate logistic regression models revealed that FESC was an independent risk factor for PAD in T2D patients. When combined with ABI, the AUC values for diagnostic, positive predictive, and negative predictive value of FESC for PAD were 0.907, 0.733, and 0.920, respectively, whereas the specificity and sensitivity were 0.914 and 0.750, respectively. Those findings allowed the conclusion that FESC, in combination with ABI, can be exploited for accurate early diagnosis of PAD.

Another study in 2022 was done by Oh et al. [57], which analyzed the clinical effectiveness of SUDOSCAN^®^, alone or in combination with the MNSI, in the screening of DPN in people with T2D. The study considered a population of 144 Korean T2D patients (85 males, mean age 59.4 ± 8.9 years), and the presence of DPN was assessed through a nerve conduction study (NCS). In addition, all participants underwent feet ESC assessment through SUDOSCAN^®^, the MNSI-Questionnaire (MNSI-Q), the MNSI-Physical Examination (MNSI-PE, consisting of inspection of feet, ankle reflexes, and vibration sensation assessed through a 128 Hz tuning fork) and the 10 g monofilament test. As for the ability of feet ESC to distinguish DPN, the authors found an AUC-ROC equal to 0.663 and identified 56 μS as the optimal cut-off value. Comparing capability of distinguishing the presence of DPN, the model including MNSI (MNSI-Q and MNSI-PE) plus SUDOSCAN^®^ showed higher AUC than the model with MNSI only (0.717 vs. 0.638, *p* = 0.011); the model MNSI plus SUDOSCAN^®^ achieved sensitivity and specificity equal to 70.0% and 66.3%, respectively. Considering the SUDOSCAN^®^ only model, results were comparable to those obtained with the MNSI plus the 10 g monofilament test or with the MNSI only model. The authors concluded that SUDOSCAN^®^ plus MNSI might be an acceptable option for the screening of DPN in Korean patients with T2D in real clinical settings.

One last study in 2022 was that from García-Ulloa et al. [58]. In a cohort of 2243 patients with recent diagnosis of type 2 diabetes, the study assessed the sudomotor function through ESC using the SUDOSCAN^®^ device. The alterations in ESC detected by SUDOSCAN^®^ were investigated. Specifically, sudomotor dysfunction was evaluated according to the measurement of the ESC in the feet, with thresholds derived in previous studies, i.e., ESC > 70 μS indicating no dysfunction, ESC in the 70–50 μS range indicating moderate dysfunction, and ESC < 50 μS indicating severe dysfunction. Thus, the study cohort was divided into two groups, the group of those with normal sudomotor function (ESC > 70 μS) and the group of those with sudomotor dysfunction. (ESC ≤ 70 μS). The study also aimed to evaluate the diagnostic accuracy of SUDOSCAN^®^ in comparison to monofilament and tuning fork tests for detecting DPN. Additionally, the study explored the association of DPN with some metabolic and clinical variables. It was found that 27.6% of the patients had sudomotor dysfunction, whereas the monofilament and/or tuning fork tests were abnormal (i.e., they identified DPN) in 28.9% of the patients. The SUDOSCAN^®^ had an AUC-ROC of 0.495 for DPN identification, with sensitivity of 24% and specificity of 71%. In logistic regression analysis, age, diastolic blood pressure, heart rate, plasma glucose, albuminuria, and beta-blockers and fibrate use were associated with sudomotor dysfunction.

In 2023, a study by Calikoglu et al. [59] aimed to investigate the SMD in some detail, studying patients with both type 1 diabetes (T1D) and T2D, as well as patients with prediabetes (PRED) and nondiabetic healthy control subjects (CNT), for a total of 690 participants. Authors noted that dry foot skin, as a result of SMD, seems to precede DPN, and it is associated with an increased risk of diabetic foot ulcers. On the otherhand, it was emphasized that SMD can be easily assessed by ESC. First, a 5.07/10 g Semmes–Weinstein monofilament was used on the hallux plantar surface and the heel center, whereas vibration sensation test was performed using a 128 Hz tuning fork placed over the dorsum of the great toe on the bony prominence of the bilateral distal interphalangeal joint. A questionnaire (MNSI) about foot sensation (pain, numbness, temperature sensitivity) was then used, along with one about general asthenia and one about peripheral artery disease. Two or more positive responses were assumed to be an indication of DPN presence. ESC values were assessed using the SUDOSCAN^®^ device. Participants underwent a 3 min scan in standing position. A low direct current (DC) voltage (<4 V) was applied incrementally, yielding a current around 0.2 mA. The ratio of the current and the DC stimulus yielded the ESC measure, which was then normalized to the body mass index (BMI). In addition, the authors assumed the mean ESC/BMI minus one standard deviation in the CNT group as the threshold for SMD. Accordingly, the prevalence of SMD was up to 59% in T2D. It was also found that mean ESC/BMI was lower in patients with DPN than in those without.

One further study in 2023 was published by Chiu et al. [60]. The aim of the study was to assess sudomotor function as a marker of peripheral neuropathy/polyneuropathy in patients both with and without diabetes (344 and 356, respectively) across various stages of chronic kidney disease (CKD). Abnormal ESC, i.e., presence of sudomotor dysfunction, was defined as hands < 40 μS or feet < 50 μS, as derived by the SUDOSCAN^®^ device. Of note, it was indicated that SUDOSCAN^®^ also incorporates built-in algorithms that integrate ESC with additional biometric data to provide a CAN score and a nephropathy score estimating the risk of CKD. In the study, clinical neuropathy scores, including MNSI and DN4 questionnaires, were also collected. It was found that hands and feet ESC decreased with CKD progression, and diabetic patients had lower hands and feet ESC than non-diabetic patients as CKD progressed. Furthermore, in a subgroup of 421 patients, the sudomotor function was found to correlate with the clinical neuropathy scores.

**Table 1:** Main information about the studies with the use of SUDOCAN^®^, Impeto Medical SAS, France *.

Publication Year	Number and Type of Patients	Main Outcomes Related to Skin Conductance/Bioimpedance Measurement	Reference Number
2010	133 T2D, 41 healthy	ESC significantly lower in diabetic patients (hands: 53 ± 16 µS, feet: 67 ± 14 µS) compared to controls (hands: 68 ± 16 µS, feet: 80 ± 7 µS); sensitivity of 75%, specificity of 100%, and AUC of 0.88.	[36]
2012	265 T2D	Lower ESC associated with increased neuropathy symptoms (MNSI A) and higher scores on physical abnormalities (MNSI B); sensitivity of 73%, specificity of 62%. Lower ESC also associated with increased VPT and abnormal CAN results.	[37]
2013	20 T1D, 63 T2D, 210 HC	Hands and feet ESC decreased in DPN patients compared with HC and DM without DPN. ESCs correlated with clinical, somatic, and autonomic measures of neuropathy and with pain scores. ROC for hands and feet ESC showed AUC values of 0.86 and 0.88 and sensitivity of 78%, and specificity of 92% for feet. Test–retest reliability for feet showed a correlation coefficient of 0.814 and a mean percentage change of 1.15%.	[38]
2014	55 DSP, 42 healthy	DSP patients showed lower ESC values with respect to healthy controls. ESC agreed with IENFD on DSP diagnosis in 58% of cases and in 67% of cases with NCE.	[39]
2015	296 DM	28.4% of the participants had moderate SMD while 25.3% had severe. An ESC decrement was observed at the feet as the severity of peripheral neuropathy and the risk of ulceration increased.	[40]
2015	45 T1D (24 with DPN and 21 without DPN), 25 healthy	ESC was lower in DPN patients with respect to no-DPN patients and controls. No differences were observed between no-DPN patients and controls. ESC taken at the feet had a sensitivity of 87.5% and specificity of 76.2% for DPN identification (cut-off 77.0 μS). For CAN identification, ESC sensitivity and specificity were lower (60.0% and 76.0%).	[41]
2016	100 healthy children (age 2–17 years, 45 males and 55 females)	No correlation between ESC and age was reported. Normative ESC values for children (80.1 ± 6.6 µS and 81.9 ± 6.2 µS for hands and feet) were similar to those reported for adults. Results indicate that maturation of the sudomotor nerve happens during childhood.	[42]
2016	1350 healthy	Median ESC values were 76 μS (62–86 μS) for hands and 83 μS (75–89 μS) for feet. ESC had weak correlation with age (−0.2) and no correlation with BMI. African American, Indian, and Chinese individuals showed significantly lower ESC values with respect to the white population.	[43]
2017	523 T2D (110 with DPN, 413 without DPN)	Abnormal ESC values (<60 μS) had higher prevalence in the DPN group (85%) with respect to VPTT (72%), and DNS (54%). As a diagnostic tool, ESC showed a higher sensitivity with respect to VPTT (85% versus 72%) and a higher AUC (0.88 versus 0.84).	[44]
2018	47 T2D (27 with DPN and 20 without DPN), 16 healthy	ESC outperformed QSART for DPN diagnosis, with an AUC not lower than 0.70 for feet and hands ESC. Sweat volume and response latency measured from leg and upper limb QSART showed AUC values that overall were not higher than 0.53. ESC showed a weak correlation (significant) only with leg sweat volume.	[45]
2018	265 T2D	ESC at hands and feet significantly correlated with total BLB (0.165 and 0.122) and uncorrelated BLB (0.172 and 0.175), but not with correlated BLB. Higher total and uncorrelated BLB levels showed independent association with higher ESC values.	[46]
2018	217 healthy	Normative ESC values for Indian adults were equal to 68.9 ± 13.1 and 71 ± 12.9 µS for the hands and feet, respectively. A weak but significant inverse correlation was found with age, whereas no significant correlation was observed with BMI. No significant difference was observed with gender.	[47]
2018	523 T2D (with DPN)	Sudomotor dysfunction is highly prevalent in patients with T2D, especially in those with DPN. There was a strong positive association between DPN subjects and sudomotor dysfunction (ESC < 60 μS).	[48]
2018	63 with peripheral neuropathy (DPN or other etiology)	A significant though weak correlation was found between ESC and skin-biopsy SGNFD/IENFD. Several patients with an abnormal ESC had a normal SGNFD, thus suggesting that some unmyelinated fibers are present but not functional.	[49]
2018	236 DPN	Instrumental evaluation through sural nerve conduction velocity and amplitude and through SUDOSCAN^®^ for sudomotor function were used to evaluate DPN prevalence, which was 51.5% for the first and 38.2% for the second.	[50]
2019	47 NGT, 50 PRED, 100 T2D	DEC had high specificity (up to 95%) when compared to MFT, EMG, NDS, and DN4, each assumed to be a possible reference for DPN identification, but the sensitivity was low.	[51]
2019	221 T2D	Abnormal ESC (<60 μS and 70 μS, respectively) values at hands or feet have a sensitivity of 97% and a positive predictive value (87%) when detecting neuropathy in patients with longer diabetes duration	[52],
2020	31 with newly diagnosed T2D, 33 with longer duration T2D and DPN, 20 with DFU, 81 PRED (control)	ESC asymmetry tended to increase from newly diagnosed T2D to DFU. ESC feet asymmetry provided an AUC of 0.955 in discriminating DFU, thus acting as an early marker. Considering a cut-off value of >9.5%, 80% sensitivity and 91% specificity were obtained.	[53]
2020	102 DM	ESC and the COMPASS 31 questionnaire had sensitivities of 83% and 75% for CAN (though with somehow lower specificity) and specificities of 67% and 65% for DPN (though with somehow lower sensitivity), respectively; when combining ESC and COMPASS 31, sensitivity for CAN increased to 100%, and specificity for DPN increased to 89%.	[54]
2022	515 T2D	The abnormal group (ESC < 60 μS) was found to have a 5.63-fold increased likelihood of having DPN than the normal group (ESC > 60 μS). Hands and feet ESC is significantly and negatively correlated with VPT. Both hands and feet ESC provided AUC values equal to 0.71; cut-off values were 73 μS (sensitivity 61%, specificity 71%) and 61 μS (sensitivity 79%, specificity 65%) for hands and feet ESC, respectively.	[55]
2022	183 T2D (36 uncomplicated, 103 with DPN, 44 with PAD)	When combined with ABI, feet ESC for prediction of PAD showed an AUC of 0.907, positive predictive and negative predictive value of 0.733 and 0.920, and specificity and sensitivity of 0.914 and 0.750, respectively.	[56]
2022	144 T2D	Feet ESC identified DPN with an AUC equal to 0.663 and identified 56 μS as the optimal cut-off value. MNSI plus SUDOSCAN^®^ showed a higher AUC than the model with MNSI only (0.717 vs. 0.638); the model MNSI plus SUDOSCAN^®^ achieved sensitivity and specificity equal to 70.0% and 66.3%, respectively (cut-off value equal to 5).	[57]
2022	2243 T2D	SUDOSCAN^®^ had an AUC of 0.495 for DPN identification, with sensitivity of 24% and specificity of 71%; age, diastolic blood pressure, heart rate, plasma glucose, albuminuria, beta-blockers, and fibrate use were associated with sudomotor dysfunction (ESC ≤ 70 µS).	[58]
2023	80 T1D, 438 T2D, 88 PRED, 84 healthy	ESC/BMI was lower in DPN patients; ESC/BMI was lowest in T2D and highest in healthy subjects, prevalence of SMD was up to 59% in T2D; retinopathy, female gender, and e-GFR were associated with SMD.	[59]
2023	344 with DM, 356 without DM	DM had lower ESC than non-DM patients; ESC decreased with CKD progression; in a subgroup of 421 patients, ESC correlated with clinical neuropathy scores.	[60]

* ABI: ankle-brachial index; AUC: area under the curve; BLB: bilirubin; BMI: body mass index; CAN: cardiovascular autonomic neuropathy; CKD: chronic kidney disease; DEC: dermal electrochemical conductance (synonym of ESC); DFU: diabetic foot ulcer; DM: diabetes; DNS: diabetic neuropathy symptom; DPN: diabetic polyneuropathy/peripheral neuropathy; DSP: distal symmetric polyneuropathy; e-GFR: estimated glomerular filtration rate; ER: electrodermal resistance; ESC: electrochemical skin conductance; HC: healthy controls; IENFD: intraepidermal nerve fiber density; IFG: impaired fasting glucose; IGT: impaired glucose tolerance; MNSI: Michigan Neuropathy Screening Instrument; NCE: nerve conduction examination; NGT: normal glucose tolerance; PRED: prediabetes; QSART: quantitative sudomotor axon reflex test; SGNFD: autonomic sweat gland nerve fiber density; SMD: sudomotor dysfunction; PAD: peripheral artery disease; T1D: type 1 diabetes; T2D: type 2 diabetes; VPT: vibration perception threshold; VPTT: vibration perception threshold test.

## 4. Studies with Different Sensors and Devices

Summary information about the studies analyzed in this section is reported in Table 2.

The pioneering study by Tackmann and Lehmann in 1980 [61] aimed to compare data of motor and sensory nerve conduction in diabetic neuropathy, giving special attention to early changes in sensory nerves with still normal conduction velocity. The study included 33 diabetic patients aged 22–73 years with different characteristics. In fact, four patients revealed muscle weakness, absent tendon reflexes, and pain, 12 showed decreased tendon reflexes and slightly affected sensation, and 17 showed no signs of peripheral nervous system abnormalities. Motor nerve conduction studies were done for all patients by applying supramaximal stimuli using bipolar surface electrodes and recording evoked muscle action potentials with surface electrodes. Nerve conduction velocities were also measured. Surface electrodes were placed around fingers, whereas Teflon-covered steel needles were placed near the sural nerve at the lateral malleolus. Both single and repetitive (train) impulses stimuli were applied, and isolated steel needles located near the nerves were used for recording. The authors found that the involvement of motor nerves was more prominent in the peroneal nerve than in the median nerve. In all cases where motor nerves were altered, involvement of sensory potentials was noted, and the degree of affection was larger in sensory than in motor fibers. As expected, application of repetitive stimuli allowed more sensitive tests in the estimation of peripheral nerve function.

Ionescu-Tírgovişte et al. [62] in 1985 investigated electrophysiological parameters and perception threshold for a deep electric stimulus applied in the shanks, as well as skin electric potential. The aim was to determine their association with the functional condition of both the somatic and autonomic nervous systems in the lower extremities. The study included 35 diabetic patients, 14 type 1 and 21 type 2, comprising 24 men and 11 women with a mean age of 56 ± 17 years. Eighteen of these patients showed signs of peripheral neuropathy, while 17 did not, and they were compared with a control group of 10 non-diabetic subjects composed of six men and four women, aged 53 ± 17 years. Cutaneous electric potentials were assessed via a 3466 Hewlett Packard voltmeter, while the perception threshold was evaluated from an electrical stimulus of 1 cm deeply applied through a bipolar impulse generator used in electroacupuncture, by increasing the current intensity progressively until the patient perceived a tingling sensation at one of the two stimulated points. The control group exhibited the highest values of cutaneous electric potential, and the neuropathic group exhibited the lowest, indicating that lower values could signify an advanced stage of peripheral autonomous diabetic neuropathy. Interestingly, diabetic individuals without clinical neuropathy had skin electric potentials resembling those of non-diabetics. However, the study showed marked standard deviations in all cases. This variability was attributed to the specific methodologies employed or to physiological factors that influence the electrical properties of the skin. Electric potential values at acupuncture points were significantly higher than those in indifferent areas—about 1 cm distant from acupuncture points—across all cases, reconfirming the acupuncture points as the “electrical windows of the skin.” Significantly higher perception threshold values were found in patients with clinical signs of neuropathy, suggesting that in these cases, the sensory nervous alterations affected both vegetative and somatic nervous systems. Furthermore, a significant inverse relationship was observed between skin electric potentials and perception thresholds, implying that increased perception thresholds lead to decreased cutaneous electric potentials, both at acupuncture points and indifferent areas. This study underlined the complex interplay between the somatic and autonomic nervous systems in diabetics, suggesting that electrophysiological measurements can provide valuable insights into the extent of neuropathic alterations.

Again in 1985, Brismar et al. [63] described a method for excitability analysis in peripheral neuropathy. The aims were finding a method to measure the threshold range that could be used in clinical context and determining the abnormal threshold range in peripheral neuropathy. For this purpose, the median nerve and the nerves proximal to entrapment were analyzed. The median nerve was stimulated with a monopolar approach with the cathode located over the median nerve at the wrist and the anode on the dorsum of the wrist, and the excitability was studied with 0.2 ms duration pulses. The muscle action potential was recorded with metal disc electrodes placed over the thenar eminence and distally on the thumb. Motor and sensory conduction velocities were assessed in the median, peroneal, and sural nerves with surface electrodes. The study showed that the range of the electrical thresholds in nerve fibers was higher in patients with suspected neuropathy, of either uremic or diabetic origin. Of note, some of those patients had normal nerve conduction velocity even in all studied nerves, except for the sural nerve, where velocity was decreased. In addition, the voltage required for nerve excitation was increased in those patients.

In 1987, Prună et al. [64] conducted a preliminary study to explore the efficacy of their “neurovegetative reactometer” device in assessing autonomic dysfunction in diabetic patients. Specifically, the research aimed to identify a non-invasive, reliable method for early detection of diabetic neuropathy. The study involved 39 diabetic patients (20 males and 19 females, aged 55 ± 10 years, with a diabetes duration of 6 ± 4 years, including 12 type 1 and 27 type 2 diabetic patients) with and without clinical neuropathy. These participants were compared to 24 apparently healthy controls. The neurovegetative reactometer evaluated sympathetic activity of the skin by measuring changes in skin electrical resistance (ΔR/R) and the latency of response to external stimuli (in seconds) using a dual-channel self-balancing impedance reactometer. The stimuli were acoustic, optical, or electric impulses of short duration, triggering a neurovegetative reaction reflected in the bioelectric parameters of the skin, primarily through sweat gland activity. Measurements were performed in a controlled environment using a pair of surface electrodes placed on the skin, employing a self-balancing bridge in a sinusoidal alternating current to detect changes in electrodermal resistance. The results after acoustic stimuli showed a significant difference between diabetic and control subjects, in both ΔR/R and latency (*p* < 0.001), indicating decreased skin sympathetic activity and increased response time in diabetic patients, likely due to autonomic denervation and slower nerve conduction velocities. This suggested a decrease in sweat gland activity and alterations in axon membrane function among diabetic patients.

In 1990, Ionescu-Tirgoviste et al. [65] employed their neurovegetative reactometer to measure electrodermal response in order to non-invasively detect autonomic dysfunction in diabetes. The 1990 study included 60 diabetic patients (32 females and 28 males) with an average age of 46.8 ± 11.8 years, comprising both insulin-dependent and non-insulin-dependent individuals, with an average diabetes duration of 8.6 ± 4.6 years. Among them, 27 patients presented clinical signs of neuropathy, such as cardiac parasympathetic or vascular sympathetic neuropathy, while 28 subjects exhibited electroperception and vibration thresholds above normal values, indicative of somatosensory neuropathy. These participants were compared against a control group of 50 non-diabetic subjects (22 females and 28 males, average age 47.5 ± 14.1 years), all apparently healthy. The evaluation parameters included skin electrical resistance relative variation (ΔR/R), latency (LT, i.e., the time interval between the stimulus application and the onset of ΔR/R at both the palm and foot), and the amplitude of response as the rate of time change along with autonomic conduction velocity, calculated by the height/LT ratio. The device employed a phase-sensitive detection and alternating current for better noise rejection, enhancing measurement sensitivity. Significant differences (*p* < 0.001) were found between diabetic and control groups across all studied parameters, indicating altered skin sympathetic activity in diabetic patients. Specifically, diabetic patients exhibited decreased ΔR/R, suggesting reduced sweat gland activity and possibly axon membrane function alterations. The study identified three stages of neuropathic progression based on electrodermal response patterns: a decrease in response at the foot but maintained at the hand, an absence at the foot with a decrease at the hand, and an absence at both sites. Interestingly, even some diabetic patients without clinical neuropathy showed decreased foot responses, highlighting the sensitivity of electrodermal activity as a possible indicator of small fiber dysfunction. Furthermore, the amplitude of the evoked electrodermal response was highlighted as a critical and reproducible parameter for diagnosing sympathetic autonomic neuropathy. The correlation between electrodermal response and other neurophysiological or cardiovascular indices (like electroreception and vibration thresholds or orthostatic hypotension) demonstrated the potentiality of the method in diagnosing sympathetic autonomic neuropathy.

In 2003, Nyström et al. [66] studied the efficacy of the combined analysis of information obtained by near-infrared spectroscopy (NIR) and total-body multi-frequency bioelectrical skin impedance analysis (MFBIA-body) for detecting and classifying skin modifications due to diabetes. Indeed, a high glucose level in microcirculation and capillary permeability can produce slight changes in the structure of the skin, and possible consequences of the increased permeability can be permanent nerve and tissue damage and an increased risk of diabetic foot ulcers; thus, the early detection of diabetes-related changes in skin can constitute a relevant diagnostic value. In this study, the skin reflectance spectra were measured in the hands, arms, legs, and feet of 34 diabetic men. Each diabetic subject was clinically evaluated through neuropathy grading value (NG, with 0 = no sign of neuropathy, 1 = objective sign but no symptoms, 2 = objective signs and symptoms). To reduce the variability due to gender, only men were recruited, and to discriminate between healthy and diabetic conditions, a control group of 23 healthy men was also enrolled. The impedance value from MFBIA-body measurements did not reveal differences between control and diabetic subjects, and, similarly, the matrices containing NIR spectra failed to show a clear separation of the groups. However, when the information provided by both the technologies was combined by means of principal component analysis, the discrimination between healthy, asymptomatic diabetic and diabetic with symptoms groups was evident. In summary, the results confirmed the validity of NIR and total-body impedance as a diagnostic tool for the early recognition of changes in skin due to diabetes.

In 2008, Gulbandilar et al. [67] investigated the modification of skin properties due to diabetes and its effect on static posture balance. They examined the relation between the skin resistance level (SRL, measured by a digital multimeter, DT-9923B) and the static balance standing duration (SSBD) in 30 diabetic patients (type 2) and in 30 healthy non-diabetic patients. In fact, the loss of cutaneous sensitivity in the foot plantar area of diabetic patients can induce disturbances in balance maintenance, thus increasing risk of fall. In this study, SSBD was measured on the dominant and non-dominant legs during a one-leg standing test with open and closed eyes conditions. This parameter was evaluated to verify if balance abilities in diabetic patients were affected by changes in foot skin plantar area. The modifications of the cutaneous properties in terms of conductance were measured through the skin resistance level. It was found that the SSBD and SRL for the diabetic group were significantly lower for both dominant and non-dominant legs. However, the results showed a poor relation between SRL and SSBD for the diabetic group in both the legs and for both the visual conditions (open eyes and closed eyes). The only significant correlation between the above-mentioned parameters was recognized in the closed-eyes condition for the non-diabetic group on the dominant and non-dominant one-leg standing tests.

Petrofsky et al. [68], in 2009, assessed some physiological effects arising from aging and diabetes through the analysis of electrodermal skin response to thermal stress. In fact, the ability to sweat and the limited vasodilation of blood vessels are impairments that typically affect elderly people and/or those with diabetes, since the efficiency of vascular endothelial function can be impacted by both aging and diabetes, leading to a decrease in resting blood flow and a limited response to autonomic stressors. The authors examined three groups of 15 subjects differing by age (a younger healthy and an older healthy group) and health condition (an older diabetic group). Each subject was exposed to three environment temperatures (15°, 23°, 32 °C) for 30 min, and, during this time, the sweat rate, skin blood flow, and electrodermal skin response (Biopac GSR module) were measured. All the measurements were performed by placing the sensors in the lower back and in the dorsal part of the foot. Results highlighted a significant reduction in blood flow, joined with an increased galvanic skin response and an impaired sweat ability in older diabetic subjects with respect to both of the other groups for each of the three thermal conditions. The electrodermal resistance for diabetic subjects was higher in the foot than in the back for each of the three thermal conditions, thus confirming the fact that in diabetic patients, the foot shows vascular damage earlier than other body areas.

Mueller et al. [69] developed a system in 2010 for non-invasive stimulation of cutaneous nociceptive fibers. The response elicited by such stimulation was measured in this study using pain-related evoked potentials (PREPs) to verify its validity in the earlier detection of small fiber neuropathy. In fact, small fiber neuropathy is a pathology characterized by autonomic abnormalities and sensory symptoms and is caused by a number of factors, such as autoimmune diseases, infections, autonomic neuropathies, and diabetes mellitus. The authors tested four groups of subjects differing by age (young healthy and old healthy, respectively) and pathological conditions (a group of neuropathic symptomatic patients and a group of diabetic patients without neuropathic symptoms). Electrical stimulation was administered bilaterally on the hand and foot. The physiological response measured by PREPs was analyzed by extracting specific features, such as negative peaks, latencies, and peak-to-peak amplitudes. The obtained results suggested that pain-related evoked potentials elicited by electrical stimulation can be valuable factors for the early recognition of diabetic sensory neuropathy. Indeed, diabetic patients without neuropathic symptoms showed a significant delay in latencies and a decrease in the amplitudes elicited from lower limbs, while the PREPs from upper limbs remained still comparable with those observed in healthy subjects.

In 2013, Mayrovitz et al. [70] validated a handheld portable device for the measurements of skin tissue dielectric constant. They proposed this technological solution as a more widely accessible alternative to high-frequency ultrasound technologies, which demonstrated the ability to recognize enlarged subdermal low echogenic band in individuals with diabetes. As the low echogenic band is presumed to be, at least in part, due to dermal water, the authors of the present study evaluated the presence of increased skin tissue water in persons with diabetes by means of a noninvasive measurement of the skin tissue dielectric constant (TDC) at a frequency of 300MHz in the foot dorsum (MoistureMeter, Delfin Technologies Ltd., Kuopio, Finland). The TDC value measured in this frequency band was regarded as a valuable indicator of the total tissue water. This parameter was monitored with three different-sized probes to achieve increased penetration depths (0.5, 1.5, and 2.5 mm). The monitored sites were on the anterior part of the forearm and on the foot dorsum, respectively. The population enrolled for this study was composed of 18 diabetic (DM) and 18 healthy (NODM) subjects, who were comparable with the former in terms of age, body mass index (BMI), and blood pressure. The values of TDC measured on the feet of the DM group were significantly greater than those obtained by NODM. Moreover, at a 2.5 mm depth, the TDC value obtained from the DM group was 14.8% greater than that measured from the NODM group. No statistically significant difference was recognized for the forearm TDC values between the groups. The greater TDC value obtained at the foot dorsum of the DM group can be consistent with the presence of unrecognized increased fluid content. Thus, the technological solution presented in this study was able to analyze local skin water in a rapid and non-invasive way, and the obtained results highlight the validity of this measurement method for the early recognition of changes in foot skin that may tend to cause DM-related edema.

In 2017, another study by Mayrovitz et al. [71] investigated the variations in dermal water and the level of HbA1c, with the hypothesis of an inverse relationship. A total of 50 patients affected by diabetes were recruited for this study (46 type 2 and four type 1 diabetes). Water content was measured from three anatomical sites, i.e., forearm, lower leg, and dorsum of the foot, by the TDC at 300 MHz. The device used for measures was the MoistureMeter (Delfin Technologies Ltd., Finland), which provides the ratio between the TDC and that of free space, allowing an estimation of skin water and related changes. Measures were thus dimensionless, requiring less than 10 s of probes in contact with the skin. Measurement depths were 0.5, 1.5, 2.5, and 5.0 mm below the epidermis for all the considered sites. In brief, the control unit generated a 300 MHz signal, which was reflected by the skin depending on the dielectric constant of the tissue and in turn related to the amount of free and bound water. No significant relations were observed between TDC and HbA1c values for any anatomical site or any depth, with coefficients of determination not significantly different from zero. A possible explanation could involve the potential confounding effect due to the duration of control, since HbA1c value is an indicator of a 3-month level of glucose control, but it is possible that it is not enough for describing levels of control in the investigated context.

In 2022, Tronstad et al. [72] developed a prototype instrument for skin impedance spectroscopy in the foot (big toe pulp, heel, and toe ball), with the hypothesis that DPN may lead to changes in the skin, and those changes may affect the skin impedance spectrum. Authors considered three electrode geometries (concentric ring, row, interdigitated array, and unipolar electrodes) and analyzed such electrodes in terms of sensitivity and repeatability in plantar skin impedance measurement. Based on these analyses, they eventually selected the bipolar ring electrode. Indeed, it was reported that the small gap between the inner and outer electrodes (around 0.5 mm) maintains focus of the measurement to the skin (mainly the epidermis) up to high frequencies, and the circular design minimizes the anisotropic effects, yielding improved repeatability. Practically, the electrode was designed with the Eagle tool (Autodesk Inc., San Francisco, CA, USA) and fabricated by printing silver ink on printed circuit boards (PCB) using a Voltera PCB printer (Voltera Inc., Waterloo, ON, Canada). Furthermore, a simulation tool (Comsol Multiphysics^®^) was used to assess the contribution of the different skin layers to the impedance measurement. Finally, the electrodes sock (i.e., the housing components for the electrodes attachment) was designed (FreeCAD) and then 3D printed. The instrument was then tested in a pilot study on five patients with DPN and five healthy subjects, and it was found that at the big toe, the healthy subjects had lower impedance than the DPN patients, especially in the 1–100 kHz range. It was concluded that monitoring the skin impedance spectrum may detect skin changes associated with DPN.

The 2023 study by López-Valverde et al. [73] was somewhat different than the previous ones, since they performed whole body bioimpedance analysis (BIA). Specifically, they focused on the extracellular water to intracellular water (ECW/ICW) ratio, as assessed by BIA, in patients with diabetic foot ulcers (DFU). Indeed, the authors stated that increased ECW/ICW ratio had been previously shown to be associated with malnutrition-inflammation-atherosclerosis (MIA) syndrome and mortality in patients undergoing dialysis, whereas in patients with DFU, this possible association had not yet been investigated. Therefore, the aim of the study was to fill that gap and hence investigate the prognostic value of the ECW/ICW ratio in DFU patients. To this purpose, 76 patients were recruited. Diabetes-related complications were derived from clinical records; the clinical evaluation then included tools for assessing nutritional status (CONUT) and laboratory tests again for nutritional status, inflammation, peripheral arterial disease, and kidney condition. To assess the body composition, BIA was performed with the BIA-101 Akern Systems^®^ (Italy). Results showed that the ECW/ICW ratio was a risk factor associated with early mortality in the DFU patients, within 6 months from study recruitment.

In 2024, the study by Schimpfle et al. [74] again exploited bioelectrical impedance analysis (BIA) to assess the correlation of the phase angle (PhA) parameter, as derived from the BIA (BIA: BIACORPUS RX 4004 M, MEDI CAL HealthCare GmbH, Karlsruhe, Germany), with several markers of DPN, in order to evaluate PhA as a possible diagnostic method for DPN. The study population included 104 healthy individuals and 205 patients with type 2 diabetes mellitus (T2D), among which 63 had DPN. The markers of DPN assessed in this study were the neuropathy disability score (NDS) and the neuropathy symptom score (NSS), the nerve conduction studies (NCS), the quantitative sensory testing (QST), the assay of circulating biomarkers of DPN, such as the neurofilament light chain protein (NFL), and the detection of structural nerve damage by magnetic resonance neurography (MRN). However, it was emphasized that all those methods and techniques have disadvantages, since NDS and NSS scores are of limited sensitivity and/or specificity, NCS and QST are limited by patient discomfort and time consumption, and NFL and MRN are mostly unavailable in routine clinical care. Therefore, given the advantages of BIA, the study aimed to assess the possible performance of BIA-derived PhA for detection of DPN. It was found that, assuming “confirmed clinical DPN” on the basis of some common criteria (named “Toronto Consensus Criteria”), when performing ROC analysis, PhA showed similar performance in comparison to the diagnostic methods and techniques indicated above. Thus, it was concluded that PhA is, in comparison to other methods and techniques analyzed, at least an equally good and much easier to handle marker for detection of DPN.

**Table 2:** Main information about the studies with different sensors and devices (with indication of sensor/device figure availability when reported in the original article) *.

Publication Year	Number and Type of Patients	Skin Conductance/Electrodermal Activity Measurement Approach/Device	Main Outcomes Related to Skin Conductance/Electrodermal Activity Measurement	Reference Number
1980	33 DM	Tonnies stimulator, Tektronix and Disa amplifiers, Biomac and Didac signal averages	Involvement of motor nerves more prominent in the peroneal nerve (18 patients) than in the median nerve (13 patients), with involvement of sensory potentials. Alteration to transmit frequent impulse series demonstrated in 28 of 33 median nerves and in 14 of 16 sural nerves.	[61]
1985	35 DM (14 T1D, 21 T2D), 10 HS	Digital multimeter 3466 (Hewlett Packard, USA) and bipolar impulse generator (drawings in Figure 1 and Figure 3 of the article)	Lower cutaneous electric potentials and higher perception thresholds in neuropathic patients. Significant inverse relationship between skin electric potentials and perception threshold.	[62]
1985	12 chronic renal failure, 5 DM, 5 carpal tunnel syndrome, 15 HS	ABC 80 Scandia Metric microcomputer, Medelec stimulator	Electrical threshold in nerve fibers increased in patients with suspected neuropathy of uremic or diabetic origin. Decreased velocity in the sural nerve in some patients, and voltage required for excitation of the low threshold fibers also increased in those subjects.	[63]
1987	39 DM (12 T1D, 27 T2D), 24 HS	Neurovegetative reactometer (developed by research team, Bucharest, Romania)	Significant differences in ΔR/R and latency, suggesting decreased sweat gland activity and slower nerve conduction in diabetic patients.	[64]
1990	60 DM (29 insulin-dependent, 21 non-insulin-dependent), 50 HS	Neurovegetative reactometer (developed by research team, Bucharest, Romania; drawing in Figure 1 of the article)	Significant differences in ΔR/R and latency, highlighting stages of neuropathic progression.	[65]
2003	34 DM (only men) and 23 HS	HYDRA EFC/ICF model 4200 for measuring MFBIA-body, frequency range 5 kHz-1 MHz, with 50 frequencies equally spaced on a logarithmic scale.	MFBIA-body measurement did not reveal differences between control and diabetic subjects as well as the NIR spectra. The fused information from both the technologies and processed through principal component analysis allowed discrimination between groups.	[66]
2008	30 DM (T2D) and in 30 HS	SRL (measured by a digital multimeter DT-9923B; photographs in Figure 1 of the article)	Results show poor correlation between static balance standing duration in one-leg standing (with open and closed eyes) and SRL measurements in both the experimental conditions for the DM group.	[67]
2009	15 HS (younger group), 15 HS (older group) and 15 DM	BIOPAC GSR 100B module	The galvanic skin resistance is higher for DM with respect to HS subjects in all the environment temperatures considered in this study (15 °C, 23 °C, 32 °C) and regardless of the age condition.	[68]
2010	36 HS (young), 24 HS (older), 35 DM (DPN), 22 DM (no DPN)	PREP from arms and legs (distance between a distal and a proximal at least 20 cm apart)	In DM patients with DPN, PREP latencies significantly increased, and amplitudes elicited from upper and lower limbs decreased. In DM without DPN, PREP abnormalities were recognized only from lower limbs.	[69]
2013	18 DM, 18 HS	MoistureMeter-D (Delfin Technologies Ltd., Finland), operating with a 300 MHz signal (photograph in Figure 1 of the article)	The TDC values at the feet of the DM group were significantly greater than for the HS group, while measures of TDC at the forearm were not significantly greater.	[70]
2017	50 DM (46 T2D, 4 T1D)	TDC (MoistureMeter, Delfin Technologies Ltd., Finland; photographs in Figure 1 of the article)	No relationship between TDC and HbA1c for any anatomical size or any measurement depth.	[71]
2022	5 with DPN, 5 HS	Prototype device for skin impedance spectroscopy (drawings and photographs in Figure 3 of the article)	At the big toe, the HS subjects had lower impedance than the DPN patients, especially in the 1–100 kHz range.	[72]
2023	66 with DFU	BIA (BIA-101 Akern Systems^®^, Italy)	ECW/ICW ratio was a risk factor for early (within 6 months) mortality.	[73]
2024	104 HS, 205 T2D (63 with DPN)	BIA (BIACORPUS RX 4004 M, MEDICAL HealthCare GmbH, Germany)	Assuming clinical DPN as from “Toronto Consensus Criteria,” BIA-derived PhA is an equally good marker for DPN as compared to more complex methods, such as NDS, NSS, NCS, QFT, NFL, and MRN.	[74]

* BIA: bioelectrical impedance analysis; DFU: diabetic foot ulcer; DM: diabetes; DPN: diabetic polyneuropathy/peripheral neuropathy; ECW/ICW: extracellular to intracellular water ratio; HS: healthy subjects; MFBIA: multifrequency bio-electrical skin impedance analysis; NCS: nerve conduction study; NDS: neuropathy disability score; NFL: neurofilament light chain protein; NSS: neuropathy symptom score; MRN: magnetic resonance neurography; PhA: phase angle parameter; PREP: pain-related evoked potentials; QST: quantitative sensory testing; SRL: skin resistance level; T1D: type 1 diabetes; T2D: type 2 diabetes; TDC: tissue dielectric constant. 3. Studies with the use of SUDOSCAN.

## 5. Meta-Analysis Findings

Based on the inclusion criteria, 14 papers were initially selected for the meta-analysis [38,39,40,41,44,45,48,50,51,52,54,55,57,58]. Upon reviewing the full texts, it became evident that four of the 14 studies did not clearly report the measures for both DPN and non-DPN groups [48,51,55,58]. Consequently, the meta-analysis was based on 10 studies. The total sample size comprised 1704 individuals, including 528 with DPN (neuropathic group) and 1176 without neuropathy (control group).

The studies varied in terms of race, gender, age, and methodologies used to diagnose DPN. In fact, there was notable variability in the approaches, ranging from the 10 g monofilament test and vibration perception threshold to clinical scoring systems such as the Neuropathy Disability Score, the Toronto Clinical Neuropathy Score, and the Michigan Neuropathy Screening Instrument. The meta-analysis was conducted considering the mean difference among outcomes as an aggregated effect size, given that all studies reported ESC measures on a common scale (μS). Table 3 displays the outcomes and standard errors for both groups (neuropathic and control), along with mean differences and confidence intervals (95% CI). The ESC measures considered for the meta-analysis are those at the feet.

**Figure 3:**
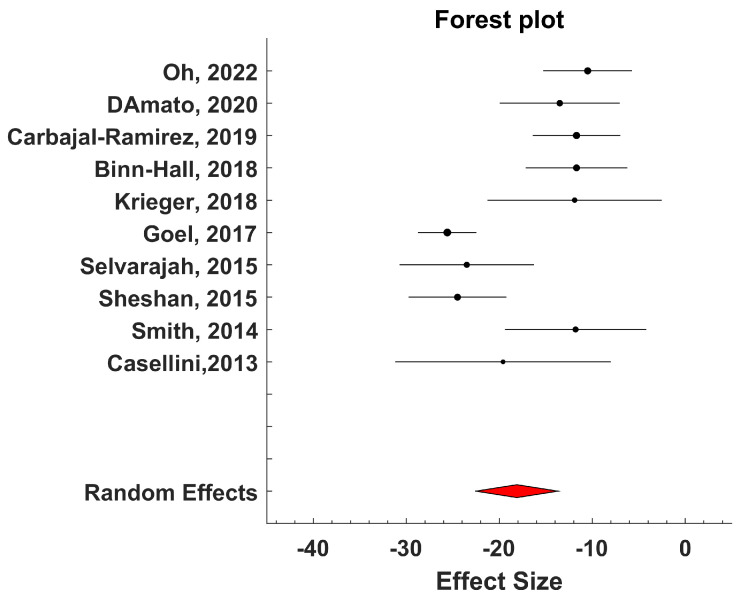
Forest plot of meta-analysis related to the evaluation of ESC measure for detecting neuropathy.

The results indicated that ESC measures were lower in the neuropathic group compared to the control group, with a statistically significant mean difference (effect size) of −18.11 (95% CI: −22.52, −13.70) (Figure 3). On the other hand, a random-effects model was employed to account for study heterogeneity, as the analysis revealed significant variability (I^2^ = 66.3%, H^2^ = 2.97, *p* = 0.002).

**Figure 4:**
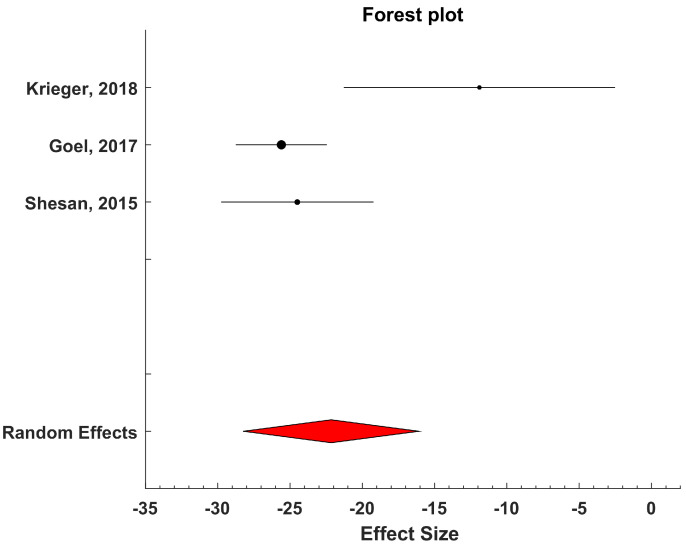
Forest plot of meta-analysis related to the evaluation of ESC measure for detecting neuropathy in the subset of studies where neuropathy was screened by Neuropathy Disability Score.

The variability in methodologies for DPN assessment may contribute to this heterogeneity. Indeed, the examined studies used different diagnostic approaches, with some studies relying only on clinical scores from questionnaires [38,41,45] and other studies reporting the use of physiological tests, like monofilament and vibration perception, in combination with clinical scores [39,40,44,50,52,54,57]. To deeply investigate our hypothesis that the method used to identify DPN could be a possible source of heterogeneity, a subset of studies was selected and further analyzed, composed of those studies for which the DPN group was detected through the Neuropathy Disability Score (NDS). For this purpose, three studies [40,44,45] among the 10 first selected were processed for this new meta-analysis. Notably, these three studies exploited different threshold values for discriminating DPN from the non-DPN condition: for two of them [44,45], the threshold for NDS was ≥ 3, while for the other [40], the threshold was ≥ 6. The analysis highlighted again a high degree of heterogeneity (I^2^ = 72.8%, H^2^ = 3.68, *p* = 0.025), although in this case, the heterogeneity was not due to the use of different methods to identify DPN, but likely to the different thresholds used for DPN identification though within the same method. It also should be noted that the studies using the same threshold [44,45] were characterized by different sample sizes of the study groups, thus possibly representing another source of heterogeneity. At any rate, despite the high heterogeneity, the meta-analysis confirmed a significant reduction in ESC values in the DPN patients, with an effect size of −22.15 (95% CI: −28.21, −16.08) (Figure 4).

**Figure 5:**
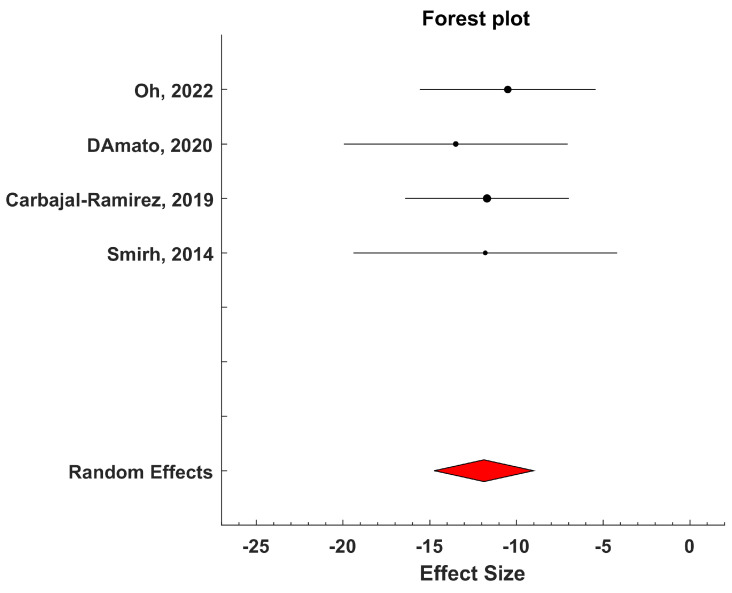
Forest plot of meta-analysis related to the evaluation of ESC measure for detecting neuropathy in the subset of studies where neuropathy was screened by Michigan Neuropathy Screening Score.

Finally, one further analysis was performed considering only those studies that used the Michigan Neuropathy Screening Score (MNSS) to identify DPN. This method, designed for outpatient use, incorporates results from a self-administered questionnaire and a physical exam involving vibration and monofilament tests. Thus, the selected subset consisting of four studies [39,52,54,57] was used for the new meta-analysis, with a MNSS threshold to identify DPN greater than 2. The findings confirmed the reduction in the ESC measure when DPN was present (Figure 5), with an effect size of −11.87 (95% CI: −14.73, −9.01). Interestingly, in this analysis, we found low heterogeneity, as indicated by the related parameters (I^2^ = 0%, H^2^ = 1, *p* = 0.09). Notably, the selected studies [39,52,54,57] were this time consistent in terms of both the methodology for DPN identification and sample size (i.e., comparable number of neuropathic subjects, ranging from 40 to 58). This corroborates our hypothesis that the heterogeneity observed in some of our meta-analyses may be due to a combined effect of the use of different methodologies for DPN identification and of the size of the patient groups in the different studies. Of course, when high heterogeneity is present, the meta-analysis results in terms of the ESC difference between patient groups have to be considered with caution.

**Figure 6:**
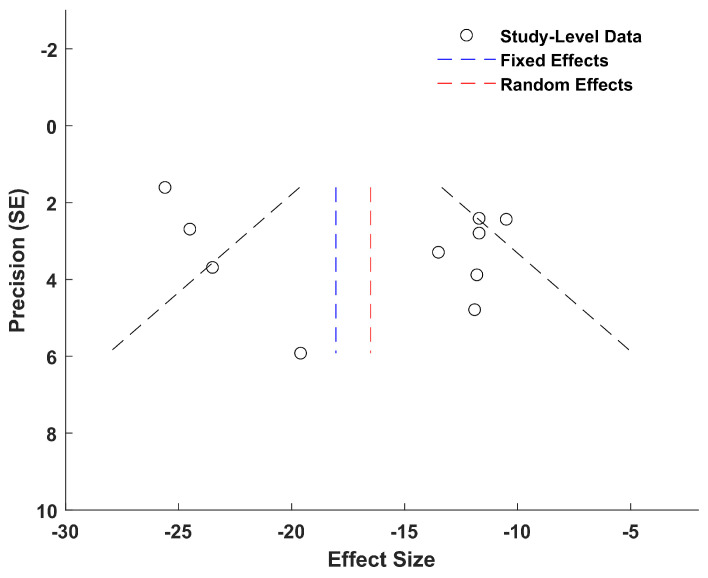
Funnel plot of meta-analysis related to the evaluation of ESC as determined by the 10 studies globally included.

The overall risk of publication bias was also eventually assessed, and the funnel plot (Figure 6) indicated limited symmetry around the random-effects model (represented by the vertical dashed line), suggesting a potential risk of bias.

## 6. Discussion

The aim of this review and meta-analysis was to analyze studies where skin conductance/bioimpedance measurements were performed for the screening of physiological/physiopathological factors related diabetic foot syndrome. The review part of the study showed that several studies demonstrated the ability of skin conductance measurements in detecting abnormalities in one of the main factors of diabetic foot syndrome, i.e., diabetic polyneuropathy/peripheral neuropathy. Of note, the majority of the reviewed studies were based on the use of a specific device, i.e., the SUDOSCAN^®^, measuring the skin conductance of feet and/or hands. For this reason, we focused the meta-analysis part of the study on the performance of this device in distinguishing between patients with and without neuropathy (the latter being determined by a reference method), and to our knowledge, our meta-analysis was the first with this aim. Interestingly, we found a clearly significant difference in the feet skin conductance of neuropathic patients as compared to non-neuropathic patients. Indeed, in the first analysis, we found a remarkable effect size (−18.11; 95% CI: −22.52, −13.70), but it was accompanied by high heterogeneity (I^2^ = 66.3%, H^2^ = 2.97, *p* = 0.002). Thereafter, we then selected a subgroup of studies based on more homogeneous methodologies (same strategy for identifying neuropathy, namely the Michigan Neuropathy Screening Score), and in that case, we found low heterogeneity (I^2^ = 0%, H^2^ = 1, *p* = 0.09), still accompanied by a notable effect size (−11.87; 95% CI: −14.73, −9.01). Therefore, we conclude that feet skin conductance is a relevant parameter for detecting diabetic foot syndrome, specifically at an early stage when there is still no presence of feet ulceration or wounds. It is also worth noting that the information derived by the meta-analysis can be used for effect size approximation and sample size calculation for future studies.

On the other hand, we believe that it is not convenient to rely on a single type of measurement for early detection of diabetic foot syndrome. Indeed, every type of measurement typically suffers from possible confounding factors that may limit its performance. Therefore, it appears convenient to combine different measurement technologies, for possible improvement of both accuracy and precision in early detection of diabetic foot syndrome. For this purpose, feet skin conductance measurement should be combined with feet skin temperature measurement, which has already been already investigated in depth (deeper than feet skin conductance), as also mirrored by several review studies [6,7,8,9,10,11,12,13]. In fact, in our opinion, the combination of feet skin conductance and temperature may be the most cost-effective solution for realizing a device aimed at early detection of diabetic foot syndrome, being sufficiently simple and cheap to be intended for personal domiciliary use and at the same time ensuring reasonably fair performance in terms of sensibility and specificity for diabetic foot syndrome detection. To our knowledge, such a home device for early diabetic foot screening is not yet available on the market.

Beyond feet skin conductance and temperature measurement, are there other types of measurement that are possibly adequate to be integrated in a home device for early diabetic foot screening? In principle, our answer is affirmative. In fact, as already discussed in one previous review study of our research group [32], feet skin humidity measurement and plantar pressure measurement may be good candidates for integration with feet skin conductance and temperature measurements in a unique device. However, it has to be acknowledged that increasing the complexity of the device may lead to difficulties in using it properly when measures are taken by the patient without supervision by health professional operators, and in addition, the cost of the device would obviously increase. Thus, both these aspects may result in being pitfalls for the development of a device intended for home, personal use. On the other hand, it may be reasonable to think about two different devices for early screening of diabetic foot syndrome: one basic device, based only on skin conductance and temperature sensors, with intended home personal use, and an advanced device that also includes other sensors (such as those for humidity and plantar pressure) but still has relatively simple use and is sufficiently cheap to be used out of the hospital context, i.e., in settings such as the general practitioner’s consulting room, the pharmacy, or even the fitness center. Of note, device solutions integrating several sensors may take advantage of modern techniques for data analysis from different sources, with special reference to those techniques based on artificial intelligence that have already proven their potential in the context of diabetic foot syndrome [26,27,28,29,30,31,32,33]. Thus, one may ask why such a device, which appears to not be extremely difficult to develop, is not yet available on the market yet. In our personal view, this may be mainly due to some underestimation by clinicians of the potential relevance of early screening for the risk of diabetic foot syndrome, even performed at home with simple and inexpensive devices (and, therefore, with some obvious limitations). As soon as the awareness of such potential relevance emerges more clearly in the clinical context, the development and market introduction of such devices will not be delayed for long.

In summary, our “take-home message” is that feet skin conductance is a relevant parameter for early detection of diabetic foot syndrome and should be combined with feet temperature in a single device intended for home personal use. On the other hand, a more advanced device that is still sufficiently simple and cheap for outpatient use may conveniently integrate other measurements, such as humidity and plantar pressure. In consideration of the severity of diabetic foot syndrome, possibly leading to feet ulceration and also amputation, such devices for early screening would be of remarkable usefulness.

## Figures and Tables

**Table 3:** Mean values of ESC measures at the feet and related standard errors (SE) for neuropathic and control groups. For each study, sample sizes for both the groups are reported (Nn and Nc, respectively).

Study	Neuropathic			Control			Mean Differences [95% CI]	Reference Number
	Mean (μS)	SE (μS)	Nn	Mean (μS)	SE (μS)	Nc		
Oh, 2022	51.5	3.32	40	62.0	0.98	104	−10.5 [−15.27, −5.72]	[57]
D’Amato, 2020	62.8	2.90	53	76.3	1.37	49	−13.7 [−19.95, −7.05]	[54]
C.-Ramirez,2019	50.9	2.15	58	62.6	1.33	112	−11.5 [−16.42, −6.78]	[52]
Binn-Hall, 2018	53.5	2.85	34	65.2	1.13	163	−11.7 [−17.17, −7.07]	[50]
Krieger, 2018	64.4	3.17	27	76.3	3.55	20	−11.9 [−21.28, −2.52]	[45]
Goel, 2017	43.7	1.78	110	69.3	0.68	413	−25.9 [−28.75, −22.52]	[44]
Selvarajah, 2015	53.5	5.10	24	77.0	1.72	21	−23.5 [−30.58, −16.27]	[41]
Sheshan, 2015	42.9	3.18	67	67.4	1.12	229	−24.39 [−29.77, −19.76]	[40]
Smith, 2014	64.0	2.96	55	75.8	2.16	42	−11.65 [ −19.40, −4.19]	[39]
Casellini, 2013	56.3	3.00	60	75.9	5.14	23	−19.6 [ −31.20, −8.00]	[38]
TOTAL (95% CI)			528			1176	−18.11 [−22.52, −13.70]	
Heterogeneity: I^2^ = 66.3%, H^2^ = 2.97, df = 9 (*p* = 0.002)								

## Data Availability

Not applicable.
